# Architectural Design Drives the Biogeography of Indoor Bacterial Communities

**DOI:** 10.1371/journal.pone.0087093

**Published:** 2014-01-29

**Authors:** Steven W. Kembel, James F. Meadow, Timothy K. O’Connor, Gwynne Mhuireach, Dale Northcutt, Jeff Kline, Maxwell Moriyama, G. Z. Brown, Brendan J. M. Bohannan, Jessica L. Green

**Affiliations:** 1 Département des sciences biologiques, Université du Québec à Montréal, Montréal, Québec, Canada; 2 Biology and the Built Environment Center, University of Oregon, Eugene, Oregon, United States of America; 3 Institute of Ecology and Evolution, University of Oregon, Eugene, Oregon, United States of America; 4 Department of Ecology and Evolutionary Biology, University of Arizona, Tucson, Arizona, United States of America; 5 Energy Studies in Buildings Laboratory, University of Oregon, Eugene, Oregon, United States of America; 6 Department of Architecture, University of Oregon, Eugene, Oregon, United States of America; 7 Santa Fe Institute, Santa Fe, New Mexico, United States of America; University of Illinois, United States of America

## Abstract

**Background:**

Architectural design has the potential to influence the microbiology of the built environment, with implications for human health and well-being, but the impact of design on the microbial biogeography of buildings remains poorly understood. In this study we combined microbiological data with information on the function, form, and organization of spaces from a classroom and office building to understand how design choices influence the biogeography of the built environment microbiome.

**Results:**

Sequencing of the bacterial 16S gene from dust samples revealed that indoor bacterial communities were extremely diverse, containing more than 32,750 OTUs (operational taxonomic units, 97% sequence similarity cutoff), but most communities were dominated by Proteobacteria, Firmicutes, and Deinococci. Architectural design characteristics related to space type, building arrangement, human use and movement, and ventilation source had a large influence on the structure of bacterial communities. Restrooms contained bacterial communities that were highly distinct from all other rooms, and spaces with high human occupant diversity and a high degree of connectedness to other spaces via ventilation or human movement contained a distinct set of bacterial taxa when compared to spaces with low occupant diversity and low connectedness. Within offices, the source of ventilation air had the greatest effect on bacterial community structure.

**Conclusions:**

Our study indicates that humans have a guiding impact on the microbial biodiversity in buildings, both indirectly through the effects of architectural design on microbial community structure, and more directly through the effects of human occupancy and use patterns on the microbes found in different spaces and space types. The impact of design decisions in structuring the indoor microbiome offers the possibility to use ecological knowledge to shape our buildings in a way that will select for an indoor microbiome that promotes our health and well-being.

## Introduction

Biologists and designers are beginning to collaborate in a new field focused on the microbiology of the built environment [1], [2]. These collaborations, which integrate perspectives from ecology and evolution, architecture, engineering and building science, are driven by a number of interrelated observations. First, it is increasingly recognized that buildings are complex ecosystems comprised of microorganisms interacting with each other and their environment [3]–[5]. Second, the built environment is the primary habitat of humans; humans spend the majority of their lives indoors where they are constantly coming into contact with the built environment microbiome (the microbial communities within buildings) [6]. Third, evidence is growing that the microbes living in and on people, the human microbiome, play a critical role in human health and well-being [7]–[9]. Together, these observations suggest that it may be possible to influence the human microbiome and ultimately human health, by modifying the built environment microbiome through architectural design.

Despite this potential, we remain in the very early stages of understanding the link between design and the microbiology of the indoor environment. A comprehensive understanding of the mechanisms that shape indoor ecosystems will entail disentangling the relative contributions of biological processes including environmental selection, dispersal, diversification, and ecological drift [10]. To date, most research has focused on understanding the influence of environmental selection and dispersal on the built environment microbiome. Environmental conditions including humidity and air temperature have been shown to influence the growth rate and survival of many microbial taxa [3], [5], [11] and correlate with the composition of bacterial communities indoors [4]. Many bacteria and fungi exhibit strong microhabitat associations and increased growth under conditions of higher humidity and in the presence of water sources, such as in kitchens and restrooms [12], [13]. The dispersal of microbes into and within the built environment also appears to have a significant influence on indoor ecosystems. The sources of microbes include those from outdoor habitats such as air and soil brought into the building via ventilation systems or carried into the building by macroorganisms [4], [14]–[16], microbes from indoor sources such as water, carpets and other surfaces within a building [13], [17], and microbes emitted from macroorganisms within the building including humans, pets and plants [18], [19]. The relative importance of these different sources of microbes indoors is not well understood, but is likely to differ as a function of space (e.g. geographic location [20]), time (e.g. year and season of sampling [15]), and building design and operation [4].

The biological processes described above can be fundamentally altered by building design. However many questions remain unanswered regarding how design aspects – such as the *function*, *form* and *organization* of a building - shape the indoor microbiome. *Function* refers to the collection of activities and uses that a building and its spaces serve. Functional requirements are translated into the variety and number of space types within a building – for example offices, restrooms, and hallways. Function is also a key determinant of the design criteria for environmental conditions including temperature, relative humidity, and light levels. *Form* refers to geometry of a building and the spaces within it, while *organization* refers to the spatial relationships among indoor spaces. Form and organization are highly interrelated and both involve design choices that influence human circulation (the source, variation and movement of people), air circulation (the source, variation and movement of air), and environmental conditions throughout a building.

**Figure 1:**
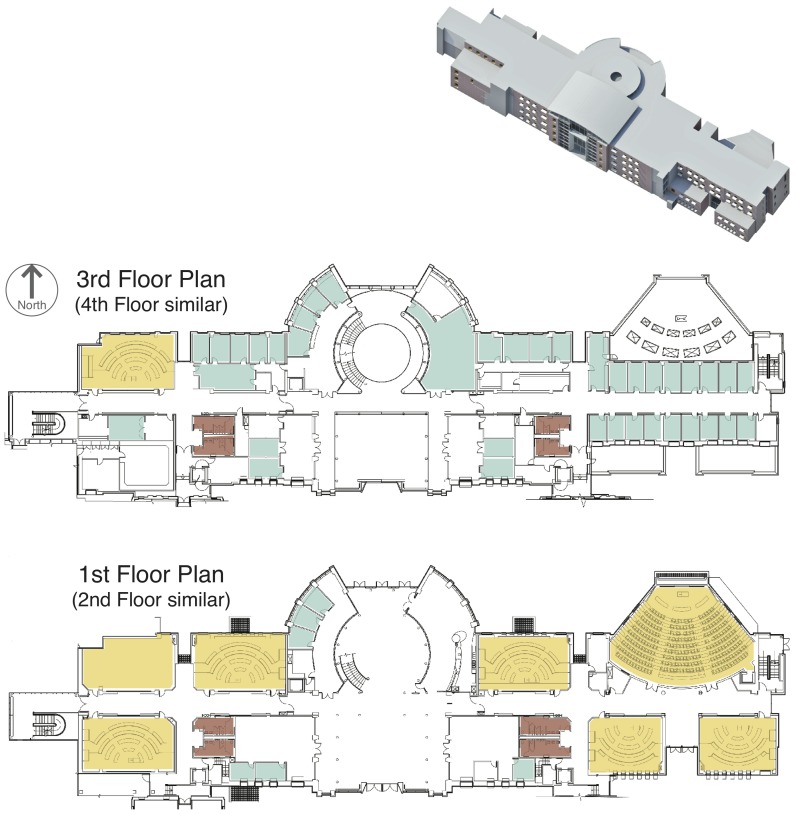
Architectural layout for two of four floors in Lillis Hall. Restrooms (brown), offices (blue) and classrooms (yellow) are shown to illustrate space type distribution throughout Lillis. The first two floors of the building are primarily devoted to classrooms and share a similar floor-plan. The 3rd and 4th floors contain most offices in the building and also share a similar floor-plan. The building has a basement and penthouse spaces; these are largely building support spaces, including mechanical rooms and storage.

To understand how design choices influence the biogeography of indoor bacterial communities, we collected microbiological, architectural, and environmental data in 155 rooms throughout a multi-use classroom and office building (Lillis Hall; Fig. 1). We focus on the bacterial communities in settled dust, because it represents an integrative record of microbial biodiversity in indoor spaces [21]. Our study addresses two overarching questions. First, at the scale of the entire building, do function, form and organization predict variation in the built environment microbiome? Second, for rooms that serve the same function (rooms that are of the same space type), which aspects of form and organization most influence the built environment microbiome?

## Methods

### Study Location

We analyzed bacterial communities in dust collected from 155 spaces in the Lillis Hall, a four-story classroom and office building on the University of Oregon campus in Eugene, Oregon, USA. This building was chosen as a study site for several reasons. Architecturally, Lillis Hall was designed to accommodate natural ventilation for both fresh air and cooling; the building is thin, allowing most rooms access to the building skin for supplying outside air directly through windows and louvers, and it has a central atrium used for exhausting air through stack ventilation. From a study design perspective, diverse space types, occupancy levels, and building management strategies were located in close proximity within the same building, making it possible to compare their relative influences on indoor biogeography.

### Architectural Design Data

Data on architectural design attributes of each space including function, form, and organization were obtained using architectural plans, field observation, and a building information model (Fig. 1). Spaces in the building were classified into one of seven *space types*. This classification system was developed for the present study based on the Oregon University System’s space type codes and definitions [40]. These categories are based on the overall architectural design and intended human use pattern for each space, and include *circulation* (e.g. hallways, atria), *classrooms*, *classroom support* (e.g. reading and practice rooms), *offices*, *office support* (e.g. most storage spaces, conference rooms), *building support* (e.g. mechanical equipment rooms, janitor closets), and *restrooms*. We measured numerous spatial and architectural attributes of each space including *level* (floor), *wing* (east versus west), *size* (net floor area), *air handling unit* (AHU) (13 different AHUs supply air to different rooms, so AHU is a categorical variable with 15 levels, one for each AHU as well as a ‘none’ category for rooms without mechanically supplied air, and a ‘multiple’ category for circulation spaces fed by multiple supply sources), and a separate binary variable denoting whether the space was only capable of being *naturally ventilated* by unfiltered outside air (e.g. via windows or louvers; 41 rooms) or by dedicated mechanical AHU supply (114 rooms).

**Figure 2:**
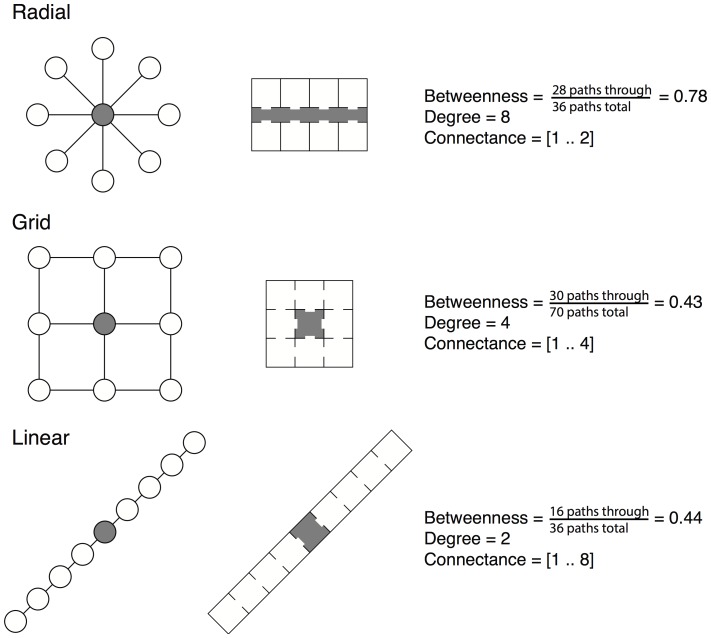
Network analysis metrics used to quantify spatial arrangement of spaces within Lillis Hall. Examples in the left column follow classic network representation, while those in the right column embody the architectural translation of networks. Shaded nodes and building spaces correspond to centrality measures [22] of *betweenness* (the number of shortest paths between all pairs of spaces that pass through a given space over the sum of all shortest paths between all pairs of spaces in the building) and *degree* (the number of connections a space has to other spaces); *connectance distance* (the number of doors between any two spaces) is a pairwise metric, shown here as the range of connectance distance values for each complete network/building. Since *betweenness* and *degree* strongly co-vary and are both measures of network centrality [22], they are considered together in some analyses.

Metrics related to *form* and *organization* were quantified using network analysis (Fig. 2) and information from building construction drawings. Spaces were considered to be spatially connected if they shared a doorway or other physical connection that would permit a person to move directly between the two spaces. The network of spatial connections among spaces was used to calculate two measures of network centrality [22], [41] for each space in the building: *betweenness*, a measure of the fraction of shortest paths among all spaces in the building that would pass through a space, and *degree*, the number of connections a space has to other spaces. The network of spatial connections between spaces was also used to define a *connectance distance* between all pairs of spaces in the building, defined as the minimum number of spaces a person would need to travel through to move between two spaces. We considered using ventilation-based distance (how much duct length separates two connected spaces) as a connectance distance, however preliminary investigation indicated that connectance distance and ventilation distance were strongly correlated.

Human use patterns are a product of functional classification, but they also dictate form and organizational attributes of building design. In this study, human use patterns for each space were estimated based on a qualitative assessment of the expected patterns of *human diversity* and *annual occupied hours* in each space. Briefly, human diversity was defined on a three-point scale, ranging from low human diversity (spaces likely to be occupied by at most a single individual during a typical day; e.g. a closet) to high human diversity (spaces likely to be occupied by numerous different individuals during a typical day; e.g. a hallway). Annual occupied hours (person-hours per year) were similarly defined along a three-point scale from low (spaces that are typically vacant or occupied at low density; e.g. a mechanical support space) to high (spaces that are frequently occupied at relatively high density; e.g. administrative offices). Both of these human occupancy variables are explained in more detail in Table S1.

At the time of microbial community sampling, ambient air temperature and relative humidity measurements were taken from each space. Relative humidity measurements were detrended using daily mean values to account for temporal changes over the sampling period.

### Biological Sampling

Sampling of dust was carried out with a Shop-Vac® 9.4L Hang Up vacuum (www.shopvac.com; #215726) fitted with a Dustream™ Collector vacuum filter sampling device (www.inbio.com/dustream.html). Dust samples were collected by vacuuming an area of approximately 2m^2^ on horizontal surfaces above head level for 2 minutes in each space. We preferentially chose these surfaces for sampling since they minimized the frequency of disturbance by cleaning, and thus likely serve as a long-term sample of airborne particles in each space [21]. All samples were collected during June 22–24, 2012. Building construction was completed in 2003, and dust has presumably been accumulating in some sampled spaces since that time.

Dust samples were stored at −80°C until DNA extraction. Dust was manually extracted from filters, and used for DNA extraction. Whole genomic DNA was isolated from samples using MO BIO PowerLyzer™ PowerSoil® DNA Isolation Kit (MO BIO, Carlsbad, CA) according to manufacturer’s instructions with the following modifications: bead tubes were vortexed for 10 min; solutions C4 and C5 were substituted for PW3 and PW4/PW5 solutions from the same manufacturer’s PowerWater® DNA isolation kit. Bacterial communities were profiled by sequencing a ∼420 bp fragment of the V4 region of the bacterial 16S rRNA gene using a custom library preparation protocol [24]. Briefly, the protocol consisted of two PCRs. The first amplified the V4/V5 region using the primers 5′-AYTGGGYDTAAAGNG-3′ and 5′-CCGTCAATTYYTTTRAGTTT-3′ [42], [43] and appended a 6 bp barcode and partial Illumina sequencing adaptor. Forward and reverse strands were labeled with different barcodes, and the unique combination of these barcodes was used to pool samples in post-processing.

All extracted samples were amplified in triplicate for PCR1 and triplicates were pooled before PCR2. PCR1 (25 µL total volume per reaction) consisted of the following ingredients: 5 µL 5x HF buffer (Thermo Fisher Scientific, U.S.A.), 0.5 µL dNTPs (10 mM), 0.25 µL Phusion Hotstart II polymerase (Thermo Fisher Scientific, U.S.A.), 13.25 µL certified nucleic-acid free water, 0.5 µL forward primer (10 uM), 0.5 µL reverse primer (10 uM), and 5 µL template DNA. The PCR1 conditions were as follows: initial denaturation for 30 s at 98°C; 20 cycles of 20 s at 98°C, 30 s at 50°C and 30 s at 72°C; and 72°C for 10 min for final extension. After PCR1, the triplicate reactions were pooled and cleaned with the QIAGEN Minelute PCR Purification Kit according to the manufacturers protocol (QIAGEN, Germantown, MD). Amplified products from PCR1 were eluted in 11.5 µL of Buffer EB. For PCR2, a single primer pair was used to add the remaining Illumina adaptor segments to the ends of the concentrated amplicons of PCR1. The PCR2 (25 µL volume per reaction) consisted of the same combination of reagents that was used in PCR1, along with 5 µL concentrated PCR1 product as template. The PCR 2 conditions were as follows: 30 s denaturation at 98°C; 15 cycles of 10 s at 98°C, 30 s at 64°C and 30 s at 72°C; and 10 min at 72°C for final extension.

Amplicons were size-selected by gel electrophoresis: gel bands at c. 500bp were extracted and concentrated, using the ZR-96 Zymoclean Gel DNA Recovery Kit (ZYMO Research, Irvine, CA), following manufacturer’s instructions, quantified using a Qubit Fluorometer (Invitrogen, NY), and pooled in equimolar concentrations for library preparation for sequencing. Resulting libraries were sequenced in two multiplexed Illumina MiSeq lanes (paired-end 150 base pair sequencing) at the Dana Farber Cancer Institute (Boston, MA). All sequence data and metadata have been deposited in the open-access data repository Figshare (http://figshare.com/articles/Lillis_Dust_Sequencing_Data/709596).

### Sequence Processing

We processed raw sequence data with the *FastX_Toolkit* (http://hannonlab.cshl.edu/fastx_toolkit) and *QIIME* [44] software pipelines to eliminate low-quality sequences and de-multiplex sequences into samples. Sequences were trimmed to a length of 200 bp (100 bp from each paired end). We retained sequences with an average quality score of 30 over 97% of the sequence length after trimming. After trimming, quality filtering and rarefaction of each sample to 2,100 sequences to ensure equal sampling depth across samples, 329,700 sequences from 155 samples remained and were included in all subsequent analyses. We binned sequences into operational taxonomic units (OTUs) at a 97% sequence similarity cutoff using *UCLUST* [45] and assigned taxonomy to each OTU using the BLAST taxon assignment algorithm and Greengenes version *4feb2011* core set [46] as implemented in *QIIME* version 1.4. We inferred phylogenetic relationships among all bacterial OTUs using a maximum likelihood GTR+Gamma phylogenetic model in *FastTree* [47].

### Data Analysis

Statistical analysis was performed in *R* [48]. Pairwise community dissimilarity was calculated using the quantitative, taxonomy-based Canberra distance metric, implemented in the *vegan* package [49] in *R*. We also assessed the consequences of beta-diversity metric choice on our results; correlations between potential metrics are included as Fig. S1. Constrained ordinations (distance-based redundancy analysis; DB-RDA) were created utilizing the *capscale* function in *vegan*. Correlations reported on ordination axes, indicated by arrows, are based on simple linear models of environmental variables against ordination axes. Indicator taxa analysis [50] was performed using the *indval* function in the *labdsv* package [51]. Mantel and partial mantel tests were used to investigate the correlations between community and environmental distance matrices, including a distance-decay comparison, using the *mantel* function in *vegan*. Permutational multivariate analysis of variance (*PERMANOVA*) was used to test community differences between groups of samples as a way to identify drivers of variation in community structure, using the *adonis* function in *vegan*. All permutational tests were conducted with 999 permutations, and thus *p-values* are reported down to, but not below, 0.001.

## Results

### Building-scale Design Influences on the Built Environment Microbiome

**Figure 3:**
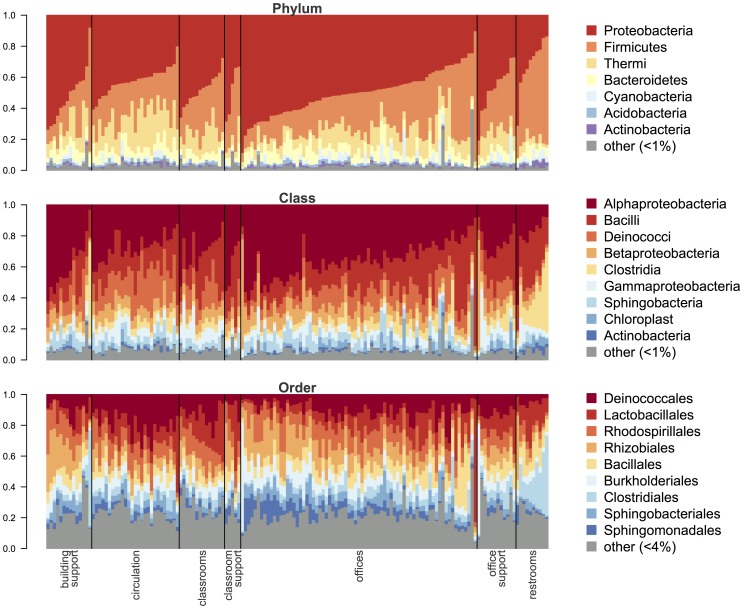
The taxonomic composition of bacterial communities sampled from dust in Lillis Hall. Samples are organized by space type, and relative abundances are shown for groups comprising more than 1% (for phylum and class level) and 4% (for order level).

Bacterial communities in dust from Lillis Hall were highly diverse. Using barcoded Illumina sequencing of 16S rRNA genes, we detected 32,964 operational taxonomic units (OTUs; defined at a 97% sequence similarity cut-off) in 791,192 sequences from 155 samples (19,403 OTUs and 325,500 sequences after rarefaction to 2,100 sequences per sample). Most of these OTUs were rare, occurring in one (49.9%) or two (13.3%) samples, and at low relative abundance (61.1% of OTUs were singletons or doubletons). However, OTUs from several taxonomic groups including Alpha-, Beta-, and Gamma-Proteobacteria, Firmicutes, and Deinococci were abundant and common in almost all dust samples we collected (Fig. 3 and Fig. S2). There were 58 OTUs belonging to these taxonomic groups that were present in 95% or more of all samples we collected. These ubiquitous OTUs were also abundant, representing 0.1% of the OTU richness but >28% of all sequences.

Spaces differing in their architectural design characteristics contained distinctive bacterial communities. Analysis of the variance in bacterial community composition explained by different factors (Table 1; PERMANOVA on Canberra distances) indicated that space type and air handling unit (AHU) explained the greatest proportion of variance (*R^2^* = 0.06 & 0.13, respectively; both *P* = 0.001). Nearly all other variables considered in this study (Table 1) were significantly correlated with biological variation as well, but explained a far smaller portion of the overall variance in microbial community structure at the scale of the building. Thus Table 1 can be seen as a potential list of building features that can, in the future, be targeted when attempting to account for microbiological variation in architectural design.

**Table 1:** Variance in biological dissimilarity among bacterial communities from all spaces, as well as just offices, (Canberra distance) explained by different variables in Lillis Hall.

Room types	Explanatory variable	*R^2^*	*P-value*
all rooms	Space type	0.06	0.001
	Air source - air handling unit (AHU)	0.13	0.001
	Building floor	0.01	0.001
	Space size	0.01	0.001
	Building wing - East/West	0.01	0.341
	Building side - North/South	0.01	0.001
	Occupant diversity	0.01	0.001
	Annual occupied hours	0.01	0.015
	Centrality (betweenness)	0.01	0.001
	Centrality (degree)	0.01	0.001
	Temperature	0.01	0.024
	Relative Humidity*	0.01	0.001
	Natural ventilation capability	0.01	0.001
offices	Air source - air handling unit (AHU)	0.07	0.001
	Building floor	0.07	0.001
	Space size	0.02	0.025
	Building wing - East/West	0.01	0.541
	Centrality (betweenness)	0.02	0.005
	Centrality (degree)	0.02	0.016
	Temperature	0.02	0.002
	Relative Humidity*	0.01	0.786
	Natural ventilation capability	0.02	0.001

Variance explained (*R^2^*) and statistical significance (*P-value*) quantified with a PERMANOVA test; since *P-values* are from permutational tests involving 999 permutations, they are only reported down to 0.001. All variables and their respective units are described in the methods section and Table S1.

* detrended using daily averages.

**Figure 4:**
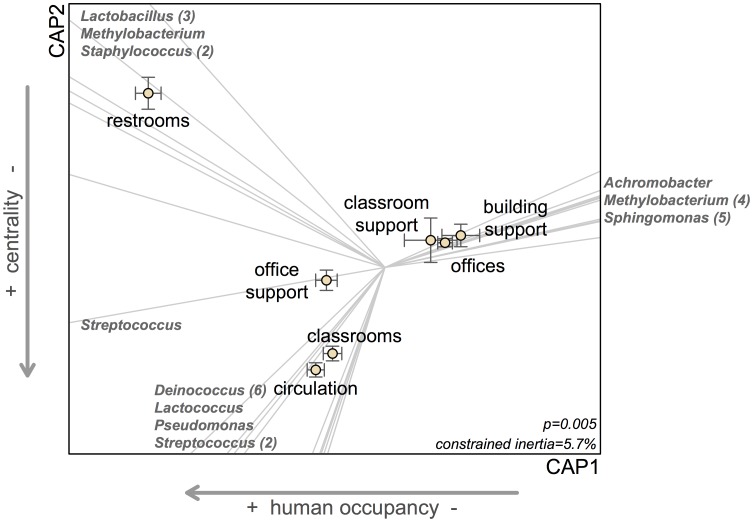
Dust communities within a building cluster by space type and are strongly correlated with building centrality and human occupancy. Points represent centroids (±SE) from distance based redundancy analysis (DB-RDA). Space types hold significantly different communities (*P* = 0.005), though this is driven primarily by restrooms. Bacterial OTUs that have the strongest influence in sample dissimilarities are shown at the margins; numbers in parentheses indicate multiple OTUs in the same genus. Centrality (along y-axis) represents network betweenness and degree; human occupancy (along x-axis) represents annual occupied hours and human diversity. All four correlates (simple linear models as a factor of ordination axis) are significant along their respective axes (all *P*<0.001).

Restrooms explained a substantial amount of the variation observed between space types; bacterial communities in restrooms were compositionally distinct from other space types (*R^2^* = 0.06; *P* = 0.001; from PERMANOVA on Canberra distances). In addition to serving a distinct function, restrooms were characterized architecturally by relatively low network centrality (quantified as network betweenness and degree [22]; network terminology outlined in Fig. 2). This is because in Lillis hall, restrooms generally only have a single door and are rarely or never on a path between any two other spaces. Restrooms also had a high diversity of human occupants (defined as a high number of different occupants throughout the day; explicit definitions of occupancy variables provided in Table S1). Indicator taxa analysis detected numerous OTUs that were associated with restrooms, predominantly belonging to taxa that are commonly associated with the human gut and skin microbiome including *Lactobacillus*, *Staphylococcus*, and *Streptococcus*. Taxa including *Lactobacillus*, *Staphylococcus* and Clostridiales were also more abundant in restrooms compared with other space types, while *Sphingomonas* were relatively less abundant in restrooms (Fig. 4).

Aside from restrooms, bacterial communities in Lillis hall tended to vary with both human occupancy and room centrality (Fig. 4). For instance hallways, which had high human occupancy and high occupant diversity (e.g., relatively many occupants and many *different* occupants throughout the day) as well as high centrality (hallways often serve as a pathway between rooms), were distinct from spaces such as mechanical support rooms and faculty offices with the opposite set of attributes (Fig. 4). While there were few statistically significant indicator taxa from individual space types other than restrooms, there was variation in the abundance of major bacterial taxa among these spaces. Taxa including *Lactococcus*, *Pseudomonas*, and *Streptococcus* were more abundant in the centrally located and highly-occupied spaces (Fig. 4), while *Achromobacter* and *Methylobacterium* were more abundant in the less central and less occupied spaces. Space types did not vary significantly in terms of their overall bacterial OTU richness or diversity (ANOVA using rarefied OTU richness and Shannon diversity; *P* = 0.2 & 0.9, respectively).

### Design Influences on the Built Environment Microbiome within a Space Type

**Figure 5:**
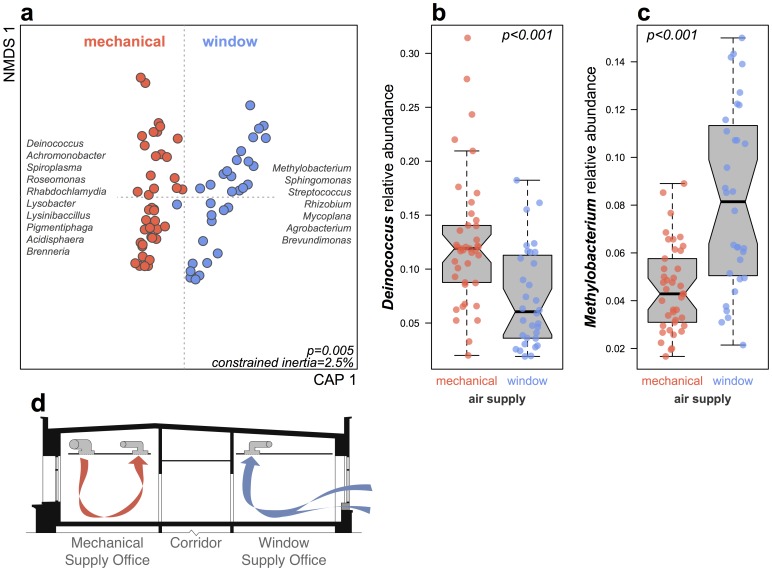
Offices contain significantly different dust microbial communities depending on ventilation source. **a)** The first axis is constrained by whether or not offices have operable window louvers (blue) or not (red). Taxon names on either side are grouped from the 25 strongest weighting OTUs in either direction. **b)** *Deinococcus* were 1.7 times more abundant in mechanically ventilated offices compared to window ventilated offices. **c)** The opposite pattern was observed for *Methylobacterium* OTUs, which were 1.8 times more abundant in window ventilated offices. Boxplots delineate (from bottom) minimum, Q1, median, Q3, and maximum values; notches indicate 95% confidence intervals. **d)** Cross-sectional view of representative Lillis Hall offices. Offices on the south side of the building (left) received primarily mechanically ventilated air, while offices on the north side of the building (right) are equipped with operable windows as a primary ventilation air source.

**Figure 6:**
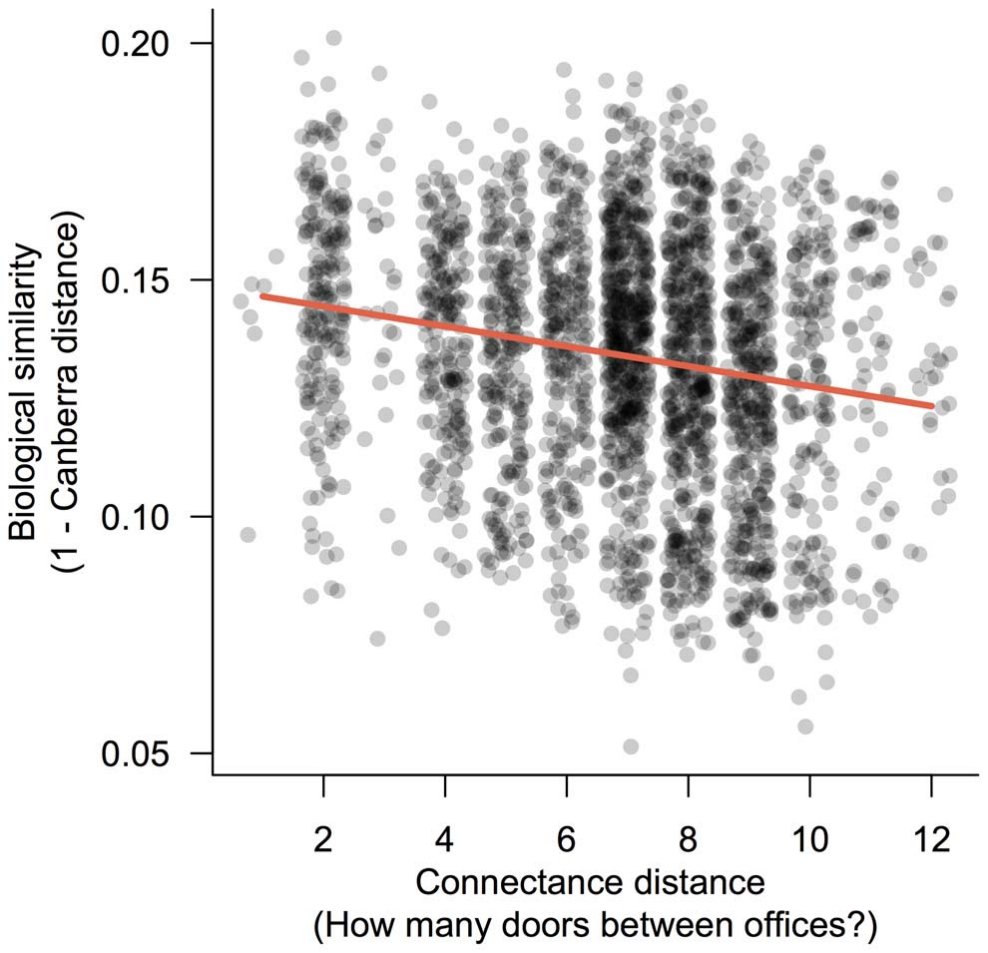
Offices in Lillis Hall show a strong distance-decay pattern. When only considering a single space type, biological similarity (y-axis; 1 - Canberra distance) decreases with connectance distance (number of intermediate space boundaries [e.g., doors] one would walk through to travel the shortest distance between any two spaces) (Mantel test; *R* = 0.189; *P* = 0.002). The same pattern was also observed at the whole-building scale (not shown; Mantel test; *R* = 0.112; *P* = 0.001).

The large number of office spaces (73 offices) made it possible to test for drivers of microbial community variation among offices. Using a single space type also allowed us to hold relatively constant several building parameters. Specifically, parameters including space size, relative humidity, and occupancy varied less across offices than across all rooms at the building-scale. Variation in bacterial community structure among faculty offices was largely explained by the ventilation source in offices, with mechanically ventilated faculty offices containing a distinctive set of bacterial taxa when compared with window ventilated faculty offices (Fig. 5; *R^2^* = 0.025; *P* = 0.005). Taxa including *Deinococcus*, *Achromonobacter*, and *Roseomonas* were associated with mechanically ventilated faculty offices, while *Methylobacterium*, *Sphingomonas*, and *Streptococcus* were more closely associated with window ventilated faculty offices. Two of the most abundant of these strongly weighting taxa, *Deinococcus* and *Methylobacterium*, when grouped by genus, show consistent abundance differences between offices with different ventilation strategies. We found a strong association between the spatial connectance distance of offices (the number of doors through which one must walk between any two spaces) versus the microbial community similarity of offices (Fig. 6; *R* = 0.19; *P* = 0.002; from a Mantel test of Canberra distance vs. spatial connectance distance). This association was also significant at the building scale, regardless of space type (*R = *0.11; *P* = 0.001).

## Discussion

In this paper we first asked: at the scale of the entire building, do *function*, *form* and *organization* predict variation in the built environment microbiome? Our data suggest that the answer is yes. In architecture, function translates to space type, which in Lillis Hall was the strongest predictor of microbiome variation throughout the building. Due to the integrative nature of architectural design, function often drives patterns in the form and organization of spaces throughout a building, and form and organization are necessarily difficult to disentangle. Although form and organization are distinct aspects of architectural design, we did not attempt to draw a distinction between them in our analyses, since nearly every building variable herein relates to both. In Lillis Hall, design choices resulted in distinct space types that greatly differed in terms of their architectural characteristics, which were related to variation in microbial community composition at the building-scale. We also focused our analyses on the most common space type in Lillis Hall: offices. Specifically, we asked which aspects of form and organization most influenced the built environment microbiome in offices. We found that network betweenness, building floor, space size, and ventilation source were the strongest predictors for microbiome variation, even after holding function constant.

Despite the microbiome variation across space types, we detected a core built environment microbiome [23] of bacterial taxa that were present in nearly every indoor space we sampled. This core microbiome was dominated by taxa including members of the Proteobacteria and Firmicutes that are commonly found in indoor dust [15], although other common indoor dust taxa such as Actinobacteria were rare in this building (c. 1% of sequences). Many of the common taxa in the indoor dust microbiome were also detected in air and surface samples from the same building [24], suggesting that resuspension and settling of microbes from these pools of potential colonists are contributing to the communities detected in dust. The synchrony among these three microbial pools (air, surfaces and dust) within Lillis Hall suggests a conserved core building microbiome. Likely sources of this core microbiome include humans, soils and plants. We found that several of the bacterial taxa most strongly associated with restrooms as well as with high occupant diversity space types, such as classrooms, are also known to be associated with the human microbiome (e.g. *Lactobacillus* and *Staphylococcus*), while bacteria in low occupant diversity space types such as faculty offices and mechanical support spaces were more indicative of outdoor environments such as soils and the phyllosphere (e.g. *Methylobacterium*).

There has been a recent debate regarding the relative importance of dispersal from outdoor sources versus the conditions within buildings for determining the structure of indoor microbial communities [16], [24], [25]. We found evidence for the importance of both types of processes: the potential for dispersal from outdoor sources (e.g. ventilation air source, natural ventilation capacity) and conditions within the building (e.g. space type, building floor, temperature and relative humidity) influenced microbial community structure. This suggests that dispersal- and niche-based explanations will be required to understand the dynamics of the built environment microbiome. As in any ecological community, the spatial and temporal scale used to define indoor communities will have a large impact on the processes that give rise to patterns of diversity [26], as will the organisms being studied (e.g. bacteria vs. fungi), and this could explain differences between our findings and those of other recent studies [16]. For example, in a large multi-use building with high occupant density such as Lillis Hall, variation in human activities and uses among space types may be the main driver of microbial community structure. In smaller buildings with lower occupant density and stronger connections to outdoor air sources (such as greater reliance on natural ventilation), dispersal from outdoors may be the more important driver of indoor microbial community structure [16].

Our study highlights network analysis as a potentially powerful tool for applying indoor ecology and biogeography to the future of building design. Our network analyses quantified patterns in the form and organizations among spaces throughout Lillis Hall. From an architectural standpoint, room arrangement within Lillis Hall follows a double-loaded corridor design, where highly-central circulation spaces (e.g. hallways) connect most rooms in the building together with few intermediate spaces (*radial* design in Fig. 2). This *radial* design strategy, compared to *linear* or *grid* designs, reduces the range of connectance distances while increasing the centrality (betweenness and degree) of circulation spaces. We found that centrality was strongly correlated with variation in microbial communities. Since increased centrality of a space inherently increases human traffic through that space, and both of these attributes predicted microbial community composition in the present study, our findings suggest that the arrangement of spaces within a building is one promising way to influence microbial community composition.

We found that design decisions can influence the ecology of microbes within a space type - for example, in faculty offices the source of ventilation air (window- or louver-supplied versus mechanically-supplied ventilation) had a large impact on bacterial community structure and the abundance of some common taxa (e.g. *Deinococcus* and *Methylobacterium*; Fig. 5). While neither of these genera are known to influence human health, the unusually high abundance of the former in building dust, and particularly in mechanically ventilated offices, gives us insight into potential selective pressures within the built environment. *Deinococcus* is a genus best known to microbiologists for the extreme oxidative stress-, desiccation- and UV-tolerance of *Deinococcus radiodurans* [27], [28]. Members of this genus are commonly found in soils, on plants, on humans, and have been detected previously in building dust and bioaerosols, but at far lower frequency than in our study [21], [29]. It is plausible that consistently low relative humidity in mechanically ventilated offices, as well as UV light from windows, created indoor environmental conditions that selected for *Deinococcus* in dust assemblages, while window ventilated offices received more frequent inputs from airborne phyllosphere and soil microbial communities, leading to higher abundances of *Methylobacterium*.

As our understanding of the drivers of indoor microbiology improve, it may be possible to design spaces that foster or inhibit the growth and accumulation of different microbial taxa in order to promote a healthier indoor microbiome. But promoting a healthy indoor microbiome will require improved information about the human microbiome and health. At this point our understanding of the drivers of microbial ecology indoors has outpaced our understanding of related health implications [1], [2], [4], [12], [13], [15], [17], [24], [30], [31]. Microbial biodiversity in the surrounding environment has been linked to human health and well-being [32]–[34], but for the vast majority of microbial taxa, we have no idea if their impact on our health is positive, negative, or neutral. Considering that the indoor microbiome represents a major potential source of microbes colonizing the human microbiome [1], [12], [13], as our knowledge about commensal microbiota expands [35]–[39], it is foreseeable that we will be able to target beneficial groups of indoor microbial taxa. Thus, while future studies will be needed to understand the health implications of indoor microbial communities, our results give clear evidence that design choices can influence the biogeography of microbial communities indoors, and thereby influence the interactions between the human microbiome and the built environment microbiome.

## Conclusion

Churchill famously stated that *“[w]e shape our buildings, and afterwards our buildings shape us.*” Humans help to direct microbial biodiversity patterns in buildings – not only as building occupants, but also through architectural design strategies. The impact of human design decisions in structuring the indoor microbiome offers the possibility to use ecological knowledge to shape our buildings in a way that will select for an indoor microbiome that promotes our health and well-being.

## Supporting Information

Figure S1 **High degree of correlation between three beta-diversity metrics.** Multivariate community analysis was carried out with the Canberra taxonomic metric; this choice results in de-emphasis of the most abundant species (as opposed to using the Bray-Curtis dissimilarity metric), and also ignores nuanced evolutionary relationships between bacterial OTUs (as opposed to using the phylogenetic Weighted UniFrac distance). While the choice of a beta-diversity metric can impact results, the three potential candidates that we explored resulted in largely the same distance between samples in multivariate space. All three metrics are bounded between 0 and 1. Pearson’s correlations (*r*) are given in the upper right panels.
(PNG)

Figure S2 **The taxonomic composition of bacterial communities sampled from dust in the Lillis Business Complex.** The relative abundance of sequences assigned to taxa at different taxonomic levels is indicated by the relative width of categories at each level. Bacterial taxonomy was visualized using Krona **(** http://sourceforge.net/projects/krona/; Ondov et al. 2011).
(PDF)

Table S1 **Explanation of occupancy variables.**
(PDF)
