# Supplementary figures and images for: Architectural Design Drives the Biogeography of Indoor Bacterial Communities
Source: PLoS One. 2014 Jan 29;9(1):e87093. doi: 10.1371/journal.pone.0087093 (PMC3906134; doi:10.1371/journal.pone.0087093)

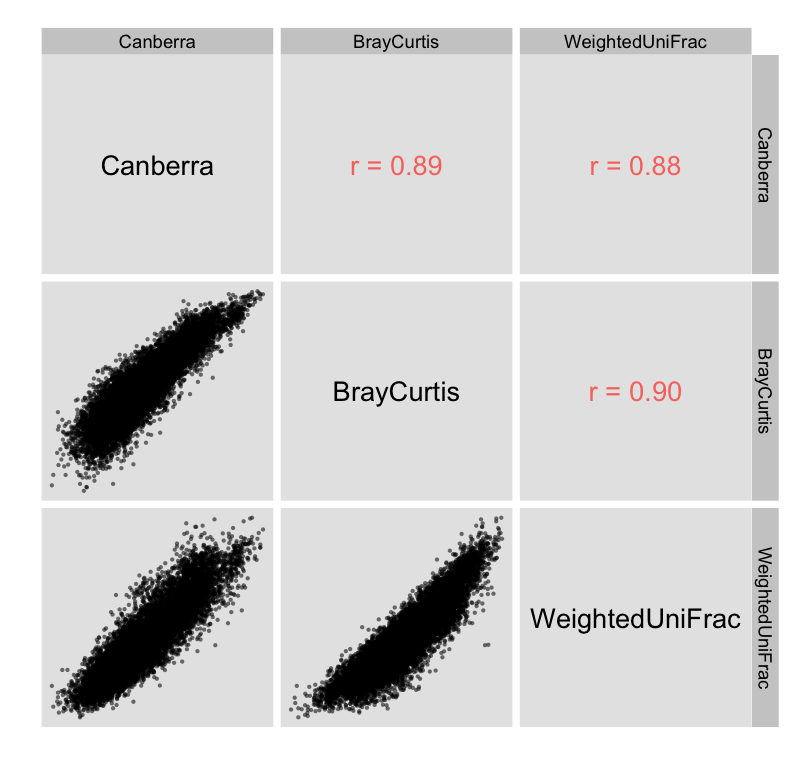

Supplement: Figure S1 — High degree of correlation between three beta-diversity metrics. Multivariate community analysis was carried out with the Canberra taxonomic metric; this choice results in de-emphasis of the most abundant species (as opposed to using the Bray-Curtis dissimilarity metric), and also ignores nuanced evolutionary relationships between bacterial OTUs (as opposed to using the phylogenetic Weighted UniFrac distance). While the choice of a beta-diversity metric can impact results, the three potential candidates that we explored resulted in largely the same distance between samples in multivariate space. All three metrics are bounded between 0 and 1. Pearson’s correlations (r) are given in the upper right panels. (PNG) [file pone.0087093.s001.png]

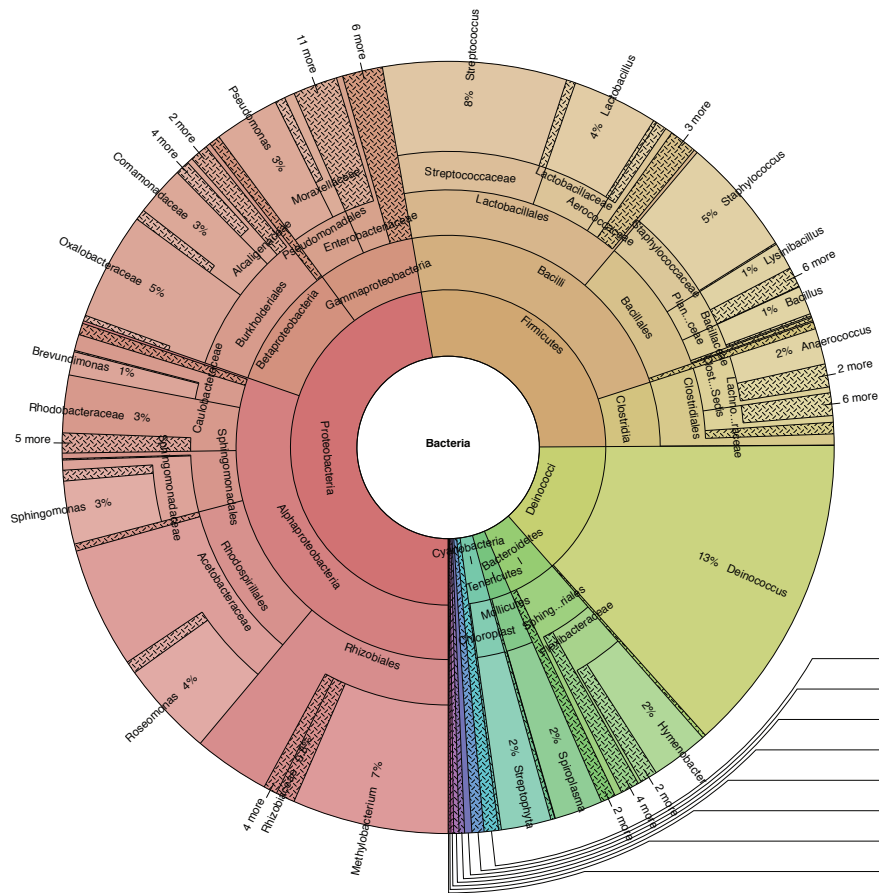

Supplement: Figure S2 — The taxonomic composition of bacterial communities sampled from dust in the Lillis Business Complex. The relative abundance of sequences assigned to taxa at different taxonomic levels is indicated by the relative width of categories at each level. Bacterial taxonomy was visualized using Krona ( http://sourceforge.net/projects/krona/; Ondov et al. 2011). (PDF) [file pone.0087093.s002.pdf]
