# Supplementary material for: Architectural Design Drives the Biogeography of Indoor Bacterial Communities
Source: PLoS One. 2014 Jan 29;9(1):e87093. doi: 10.1371/journal.pone.0087093 (PMC3906134; doi:10.1371/journal.pone.0087093)
Supplement: Table S1 — Explanation of occupancy variables. (PDF) [file pone.0087093.s003.pdf]

Supplemental Materials for:

Architectural design drives the biogeography of indoor bacterial communities.

Kembel SW, Meadow JF, O'Connor TK, Mhuireach G, Northcutt D, Kline J, Moriyama M, Brown GZ, Bohannan BJM, Green JL

Calculating human use variables

annual_occupied_hours: To estimate annual occupied hours in each space during the former 10 years of building operation, we constructed an ordinal variable with three levels (low, medium, and high) based on the following building data:

- **Intended space use:** derived from Oregon University System Facility Services Inventory Manual (http://www.ous.edu/sites/default/files/dept/capcon/files/imanual.pdf; accessed 9/2013). Many spaces were verified by site visits to ensure that intended use matched current use.
- **Classroom utilization standards:** derived from Oregon University System Facility Services Inventory Manual (above) and Oregon State Board of Higher Education minimum objectives (summarized in a 2012 University of Oregon internal study: http://uplan.uoregon.edu/plandoc/Fall2012_ClassroomSpaceUtilizationStudy.pdf; accessed 9/2013).
- **Classroom empirical usage:** combined from University of Oregon Office of Registrar data, 2011-2012 class schedules, University of Oregon academic calendar, Lundquist College of Business room occupancy schedule, periodic site visits, and 24 hour occupant surveys conducted during sampling for previous work in Lillis Hall (detailed in Meadow et al., 2013).

Resulting categories were defined as:

Bin	Annual Occupied Hours	Example
low	< 1000	mechanical spaces, 100 – 500 hrs
medium	1000 – 3000	classrooms, 1452 hrs
high	> 3000	administrative offices, 3129 hrs

Similar occupant analysis has been conducted by the authors during past consulting work for design professionals and for University of Oregon Facilities Services, Operations and Maintenance Department, and also during the development of energy simulation software (e.g., Brown et al. 1992, 1994a, 1994b, 1995, 1998a, 1994b, 1999, 2004, 2008a, 2008b)

occupant_diversity: This 3-point ordinal variable was constructed to capture the number of potential distinct individuals in a space over time. Occupant diversity was considered in all individual spaces in Lillis hall and classified based on the following criteria :

low:
Examples: closets and mechanical spaces.
Characteristics:

- Restricted access – only certain people allowed access, reducing the number of people allowed in a space.
- Not occupiable space – indicative of storage space, with a limited set of allowed visitors.
- Low occupied hours – suggests the type has less opportunity to be occupied by different individuals.
- Small floor area (<10 m^2) – inherently limits the number of people in a space.

medium:
Examples: meeting rooms, offices
Characteristics:

- Limited access – generally used only by certain groups of people.
- Often a gathering space with low frequency of use – i.e. it has high diversity events, but these happen relatively infrequently or irregularly. This distinguishes these from classrooms, or high traffic areas such as hallways.
- Small to medium floor area (10-90 m^2) – suggests there are limits to the number of people in a space at a time.

high:
Examples: classrooms, most hallways
Characteristics:

- Public access – increases the number of people allowed in a space.
- High traffic area – large numbers of people use the space or it is highly connected to other spaces.
- Gathering spaces – spaces that accommodate large groups of people.
- Medium to large floor area (>90 m^2) – inherently accommodates a larger number of people.

Spaces with clear exceptions to these criteria were considered based on their intended use (e.g., mechanical spaces with a large floor area vs. elevators with a very small floor area).

Supplementary References

Brown GZ, Meacham M, Miller P. (1992) Building Massing Intelligent Design Tool, Advanced Energy Design and Operation Technologies- Prototype 1. University of Oregon, Eugene, Oregon and Pacific Northwest Laboratories, Richland, Washington.

Brown GZ, Brambley M, Chassin D, Helou M, Raney M. (1994) Energy Module, Auto Architect, Softdesk Inc., Henniker, New Hampshire.

Brown GZ, Sekiguchi T. (1994) SIP Scheming 1.0, University of Oregon, Eugene, Oregon.

Brown GZ, Kline J, Sekiguchi T. (1995) Energy Scheming's Design Advisor. Proceedings of the 20th National Passive Solar Energy Conference, Minneapolis, MN.

Brown GZ, Raney M, Helou M. (1998) AutoCAD/Trane Energy Module, University of Oregon, Eugene, Oregon.

Brown GZ, Fremouw S, Helou M, Kline J, Peffer T, Schneider M, Sekiguchi T. (1998) The Infrared Professor (Energy Scheming 3.0). Proceedings of PLEA Conference, Lisbon, Portugal.

Brown GZ, Bjornson D, Briscoe J, Fremouw S, Kline J, Larocque P, Northcutt D. (1999) Using Simulation to Design Energy-efficient Modular Classrooms. Proceedings of American Solar Energy Society Solar '99 Conference, Portland, ME.

Brown GZ, Kessler L, Kline J, Northcutt D. (2004) Energy Zoning. Proceedings of the American Solar Energy Society Solar 2004 Conference, Washington, DC.

Brown GZ, Sekiguchi T, Kline J. (2008) Energy Scheming (version 3.2), University of Oregon, Eugene, Oregon.

Brown GZ, Chavez D, Northcutt D, Stenson J. (2008) A Comparison of Computational Fluid Dynamic Simulations and Actual Performance of a Classroom Achieving 100% Cooling Through Night Ventilation of Mass. Proceedings of the American Solar Energy Society Solar 2008 Conference, Denver CO.

Ondov BD, Bergman NH, Phillippy AM. (2011). Interactive metagenomic visualization in a Web browser. BMC bioinformatics 2011,12:385.
